# *LMNA* Determines Nuclear Morphology During Syncytialization of Human Trophoblast Stem Cells

**DOI:** 10.3389/fcell.2022.836390

**Published:** 2022-04-11

**Authors:** Yiming Wang, Hao Wu, Xiangxiang Jiang, Lei Jia, Meijiao Wang, Yin Rong, Shuo Chen, Yue Wang, Zhenyu Xiao, Xiaoyan Liang, Hongmei Wang

**Affiliations:** ^1^ State Key Laboratory of Stem Cell and Reproductive Biology, Institute of Zoology, Chinese Academy of Sciences, Beijing, China; ^2^ University of Chinese Academy of Sciences, Beijing, China; ^3^ Institute for Stem Cell and Regeneration, Chinese Academy of Sciences, Beijing, China; ^4^ Beijing Institute for Stem Cell and Regenerative Medicine, Beijing, China; ^5^ NHC Key Laboratory of Study on Abnormal Gametes and Reproductive Tract, Anhui Medical University, Hefei, China; ^6^ Reproductive Medical Center, The Sixth Affiliated Hospital of Sun Yat-Sen University, Guangzhou, China; ^7^ School of Life Science, Beijing Institute of Technology, Beijing, China

**Keywords:** human trophoblast stem cell, primary syncytialization, nuclear enlargement, lamin A, CRISPR/Cas9

## Abstract

Upon implantation, the trophectoderm differentiates into the multi-nucleated primitive syncytiotrophoblast (pSTB) through a process called primary syncytialization to facilitate maternal-fetal interactions and to establish a pregnancy. However, ethical issues and limited access to human embryos around the time of embryo implantation hinder the investigation of the detailed molecular mechanisms underpinning this event in humans. Here we established human trophoblast stem cells (hTSCs) from human blastocysts. We characterized nuclear enlargement in STB differentiated from hTSCs, which recapitulate morphological nuclear features of pSTB in human embryos. Specifically, we revealed that CRISPR/Cas9-mediated *LMNA* disruption perturbated nuclear volume during hTSCs syncytialization. Overall, our results not only provide an interesting insight into mechanisms underlying nuclear enlargement during primary syncytialization but highlight the hTSCs as an indispensable model in understanding human trophoblast differentiation during implantation.

## Introduction

Implantation is one of the most important steps in which the blastocyst invades the endometrium of the uterus toward pregnancy initiation. Until now, multiple molecules are reported to act together under strict regulation to ensure successful implantation. Failure of implantation is a major limiting element in early pregnancy and assisted reproduction. Human reproduction is surprisingly inefficient, with the majority of the losses occurring during implantation (Macklon et al., 2002). The development of trophoblast cells and their subsequent differentiation are critical to implantation (Enders and Schlafke, 1969). Human trophoblast cells are originated from an outer layer of the blastocyst, namely trophectoderm (TE) (Hertig et al., 1956). During implantation stage, some TE cells differentiated into multinucleated syncytia (primitive syncytiotrophoblast, pSTB) via a process called primary syncytialization, which is characterized by the appearance of multiple nuclei within a single cell. According to the Carnegie collections, pSTB forms at around day 8 p. f. (post fertilization). Moreover, clusters of large nuclei are contained in some of the syncytial masses (Hertig et al., 1956; Enders and Schlafke, 1969). Primitive syncytiotrophoblast also expressed the typical STB marker gene CGB (chorionic gonadotropin subunit beta) (Deglincerti et al., 2016). The structure and function of the pSTB should be precisely regulated to ensure the successful implantation. However, owing to limited access to human embryos around the time of embryo implantation and lack of a suitable investigating model in culture, the underlying mechanisms of primary syncytialization remain largely unknown.

The recent establishment of methods to *in vitro* culture human embryos at peri-implantation stage offers an unprecedented opportunity to investigate trophoblast specification, however, owing to the limited availability of human embryos and ethical issues for gene manipulation, in-depth functional studies of trophoblast differentiation during implantation are largely lacking. Several cell lines have been used to study the primitive STB formation. Trophoblast-like cells derived from human embryonic stem cells (hESCs) greatly facilitated the study of the mechanisms underlying pluripotency but not a good model because of their origin from EPI of the human embryo rather than trophectoderm (Yabe et al., 2016). Recent studies indicated that multipotent human trophoblast stem cells (hTSCs) can be derived from blastocysts and culture atop a 2D surface (Okae et al., 2018). However, whether syncytialization of hTSCs could mimic the morphogenesis of trophoblast differentiation in implanting human embryos, such as the increase of nuclear volume, has not been elucidated.

As one of the most important cellular components, the nucleus is widely believed to play critical roles in development and pathogenesis. The nucleus contains a nuclear envelope (NE), a lamina layer underneath the NE and chromatins enclosed in the center. The nuclear lamina, a meshwork at the nuclear periphery, is composed of lamin A, its splice variant lamin C (collectively lamin A/C), lamin B and their interacting proteins. *LMNA* has been strongly implicated in protecting nuclear morphology in mouse embryonic fibroblasts and mesenchymal stem cells (Swift et al., 2013; Kim et al., 2017). Abnormalities in the nuclear lamina layer are commonly observed in diseases such as laminopathies. Laminopathies are relatively rare genetic diseases including progeria syndrome (Kudlow et al., 2007), congenital muscular dystrophy (Quijano-Roy et al., 2008) and dilated cardiomyopathy (Fatkin et al., 1999). Mutations in the lamin A/C gene (*LMNA*) that encodes for the filamentous lamin A/C proteins (Hutchison, 2002; Schreiber and Kennedy, 2013) may participate in the pathogenesis of laminopathic diseases. Lamin A is developmentally regulated and their expression patterns in different tissues are correlated with organogenesis (Rober et al., 1989). Ectopic expression of lamin A in myoblasts could promote the expression of muscle-specific genes (Lourim and Lin, 1989). However, the roles that *LMNA* plays in nuclear morphology control of human trophoblast stem cells and STB are not clear.

To uncover the molecular underpins of nuclear morphology in trophoblast development during implantation, we have established hTSCs from human blastocysts. The culture system allows hTSCs to remodel nuclei enlargement during syncytialization *in vitro*, recapitulated the key hallmarks (large nuclei) of pSTB according to the sections of Carnegie collection. Our result further implicated *LMNA* as a regulator of nuclear volume in syncytialization of hTSCs. We demonstrated nuclear volume was enlarged by deletion of *LMNA*. We have determined the potential use of hTSCs in elucidating the early implantation process and trophoblast syncytialization.

## Materials and Methods

### Ethics Statement

This work was approved by the Ethics Committee of the Center for Reproductive Medicine, Sixth Affiliated Hospital of Sun Yat-Sen University (Research license 2019SZZX-008). The Medicine Ethics Committee of the Center for Reproductive Medicine, Sixth Affiliated Hospital of Sun Yat-Sen University, comprises 11 members, including experts in laws, science, and clinicians with relevant expertise. The Committee evaluated the scientific merit and ethical justification of this study and conducted a full review of the donation and use of these samples.

The informed consent process for embryo donation complied with the International Society for Stem Cell Research (ISSCR) Guidelines for Stem Cell Research and Clinical Translation (2016) and the Ethical Guidelines for Human Embryonic Stem Cell Research (2003) jointly issued by the Ministry of Science and Technology and the Ministry of Health of People’s Republic of China. The ethical and regulatory framework set forth by the Center for Reproductive Medicine, Sixth Affiliated Hospital of Sun Yat-Sen University, clearly specified that informed consent could only be obtained if eligible participants were provided with all necessary information about the study and had an opportunity to receive proper counseling. The consent clearly described the goals and related clinical procedures for the study. No financial inducements were offered for the donations.

### Thawing of Human Embryos and Removal of the Zona Pellucida

All donated embryos in this study were obtained from frozen embryos from couples who had already signed informed consent. The study employed standard clinical protocols for embryo collection, cryopreservation, thawing, and culture procedures. The human embryos used in this work were obtained from 6 days post fertilization (d.p.f.). Embryos with normal morphology and cleavage patterns were utilized in this study. Human blastocysts were thawed using Kitazato Thawing Media Kit VT802 (Kitazato Dibimed) according to the manufacturer’s instructions. The zona pellucida of each blastocyst stage embryo was removed by brief exposure to acidic Tyrode’s solution (Sigma-Aldrich). The embryos were washed in the hTSCs culture medium two times and transferred into the hTSCs medium.

### Culture of Human Trophoblast Stem Cells

Derivation and culture of human trophoblast stem cells from human embryos were performed as previously described (Okae et al., 2018). Briefly, a 4-well plate was coated with 5 μg/ml Collagen I at 37°C for at least 1 h. Thawed human blastocysts were seeded in the 4-well plate and cultured in 500 μl of hTSCs medium (DMEM/F12 supplemented with 0.1 mM 2-mercaptoethanol, 0.2% FBS, 0.5% Penicillin-Streptomycin, 0.3% BSA, 1% ITS-X supplement, 1.5 mg/ml L-ascorbic acid, 50 ng/ml EGF, 2 mM CHIR99021, 0.5 mM A83-01, 1 mM SB431542, 0.8 mM VPA and 5 mM Y27632). After 4–5 days of culture, the attached embryos were dissociated with TrypLE for 8 min at 37°C, and the single cells were passaged to a new Collagen I-coated 4-well plate and cultured in hTSCs medium. After several passages, hTSCs were routinely passaged every 2–3 days at a 1: 4-1: 6 ratios.

### Differentiation of hTSCs Into Syncytiotrophoblast and Extravillous Trophoblast Cells

Differentiation of hTSCs was performed as previously described (Okae et al., 2018). Briefly, hTSCs were grown to 80% confluence in the hTSCs medium and dissociated with TrypLE for 8 min at 37°C. For the induction of STB, hTSCs were seeded in a μ-Slide 8-well dish (IB-80826, ibidi) pre-coated with 2.5 mg/ml Collagen I at a density of 1 × 10^4^ cells per well and cultured in 200 μl of STB medium (DMEM/F12 supplemented with 0.1 mM 2-mercaptoethanol, 0.5% Penicillin-Streptomycin, 0.3% BSA, 1% ITS-X supplement, 2.5 mM Y27632, 2 mM forskolin, and 4% KSR). The medium was replaced every 2 days, and the cells were immunostained on day 6. For the induction of EVT cells, hTSCs were seeded in a 6-well plate pre-coated with 1 μg/ml Collagen I at a density of 0.5 × 10^4^ cells per well and cultured in 200 μl of EVT medium (DMEM/F12 supplemented with 0.1 mM 2-mercaptoethanol, 0.5% Penicillin-Streptomycin, 0.3% BSA, 1% ITS-X supplement, 100 ng/ml NRG1, 7.5 mM A83-01, 2.5 mM Y27632, and 4% KnockOut Serum Replacement). Matrigel was added to a final concentration of 2% shortly after suspending the cells in the medium. On day 2, the medium was replaced with the EVT medium without NRG1, and Matrigel was added to a final concentration of 0.5%. On day 4, replace the medium with EVT medium without NRG1 or KSR, and Matrigel was added to a final concentration of 0.5%. The cells were immunostained on day 6.

### Engraftment of hTSCs Into NOD-SCID Mice

Six-week-old male NOD-SCID mice were obtained from Sibeifu China and maintained under specific-pathogen-free conditions. All animal experiments were approved by the Animal Care and Use Committee of the Institute of Zoology, Chinese Academy of Sciences. 1 × 10^7^ hTSCs were resuspended in 200 μl of a 1:2 mixture of Matrigel and DMEM/F12 containing 0.3% BSA and 1% ITS-X supplement and injected into 6-week-old male NOD-SCID mice. Lesions were collected on day 7 after injection. The lesions were fixed with 4% paraformaldehyde (PFA) overnight at 4°C followed by paraffin embedding. Paraffin-embedded tissues were sectioned at 6 μm. Sections were immunostained according to standard procedures.

### Immunofluorescence

Briefly, 4% of PFA-fixed cells were permeated (30 min, 0.5% Triton X-100) and washed before adding blocking buffer (3% bovine serum albumin in PBS) for 60 min. Cells were then immunostained with primary mouse, rabbit, and goat antibodies diluted in blocking buffer as follows: OCT4 (1:200; Santa Cruz Biotechnology), SOX2 (1:200; Cell Signaling Technology), GATA3 (1:200; Abcam), TP63 (1:200; Abcam), TEAD4 (1:200; Abcam), ITGA6 (1:200; Abcam), CDH1 (1:200; Abcam), KRT7 (1:200, Zhongshan Golden Bridge Biotechnology), HLA-G (1:200; Santa Cruz Biotechnology), CGB (1:200; Zhongshan Golden Bridge Biotechnology, Mouse), SDC1 (1:200; Abcam), Lamin A (1:200, Abcam, Rabbit), and CGB (1:200, Abcam, Rabbit) overnight at 4°C. Cells were washed and incubated for 1 h at room temperature with Alexa Fluor 488 green-fluorescent or Alexa Fluor 568 red-fluorescent at 1/200 (Invitrogen) in 3% BSA. Then, the cells were washed. DAPI (15 min, 25 mg/ml DAPI in PBS) and Phalloidin (1:200, YEASEN) were added for staining. The cells were washed again before imaging. The 4% paraformaldehyde-fixed paraffin-embedded sections were subjected to immunocytochemistry with standard procedures. Antibodies to CGB (1:200; Zhongshan Golden Bridge Biotechnology) and HLA-G (1:200; Santa Cruz Biotechnology) were used. Images were acquired using a Carl Zeiss LSM 780 confocal laser-scanning microscopes with 20×, 40×, and 63× objective lens and analyzed using Zeiss LSM Image Browser software, Imaris software, and ImageJ.

### Fluorescence *In Situ* Hybridization

Follow the VividFISH™ FISH Probe kit (Vivid) manual of the manufacturer. Briefly, incubate the cells in the pre-warmed pretreatment solution (2 × SSC, 0.5% NP-40, pH7.0) at 37°C for 30 min. Then, dehydrate in 70%, 90%, 100% ethanol each 1 min, and air dry. Incubate the cells in the denaturation solution (70% Formamide, 1 × SSC, pH7.0) at 73 ± 1°C for 5 min. Dehydrate in 70%, 90%, and 100% ethanol each 1 min, then air dry. Then, pick 10 µl/chamber of FISH mixed probes into a tube, denature at 80°C for 5 mins, and place on ice. Add 10 µl of the probes to each chamber and incubate in the 42°C incubators for 20 h. After incubation, put the cells in the pre-warmed 0.5 × SSC+0.1% NP40 solution to wash out the probes. Add 2 × SSC+0.1% NP40 solution and rinse with ddH_2_O. Incubate the cells with DAPI for 15 min and wash with ddH_2_O. Images were acquired using confocal laser-scanning microscopes with a 63× objective lens.

### Single-Cell Collection

Single cells were isolated from hTSCs. The cells were incubated with TrypLE Express reagent for 8 min at 37°C and dissociated into single cells. Single cells were randomly picked with a mouth pipette in 0.1% BSA and then transferred into 0.2 ml PCR tubes (Eppendorf) containing 2.5 μl cell lysis buffer to construct the single cell library by modified Smart-seq2 and performed sequencing. The lysed cells were kept at −80°C until library preparation.

### Construction of the Single-Cell RNA-Seq Library

We used a modified Smart-seq2 protocol to construct the single-cell RNA-seq library (Picelli et al., 2014). In short, the cells were lysed to release all RNAs. Then, the mRNAs were captured with barcoded oligo-dT primers with an anchor sequence and unique molecular identifier (UMI) sequences. The mRNAs were reverse-transcribed to first-strand cDNAs. After that, the preamplification step was performed to increase cDNA yields. Finally, cDNAs from different cells were pooled together with different barcodes. After 5 cycles of PCR, the index sequence with biotin modification was added at the 3′ ends of the cDNAs. Following DNA fragmentation with an ultrasonicator, we used Dynabeads C1 (65002, Invitrogen) to enrich the 3′ cDNAs to construct the library with the Kapa Hyper Prep Kit (KK8505, Kapa Biosystems). Libraries were then sent to Novogene for quality control and sequencing. The qualified libraries were sequenced on the Illumina HiSeq XTEN platform using the 150 bp paired-end reads (PE150) strategy.

### Reads Mapping and Gene Expression Quantification

The paired-end reads of Smart-seq2 data were processed using the custom scripts of Drop-seq_tools-2.0.0. For read 2, bases 1 to 8 were tagged with cell-barcode “XC,” and bases 9 to 16 were tagged with UMI “XM.” After removing the adaptors and TSO sequences and poly(A) sequences, STAR aligner was used to align the filtered reads to the human hg38 reference genome, and reads were annotated with the GRCh38.84 annotation file. A gene expression matrix (count value) was generated with the “DigitalExpression” command function. The raw data and processed gene expression matrix data were deposited in the NCBI Gene Expression Omnibus (GEO) database with the accession GSE165131. The Smart-seq2 data from GSE109555 (Zhou et al., 2019) were also processed using the same method.

### Visualization and Clustering of the Single-Cell Data

We mainly used the Seurat3 R package to analyze the Smart-seq2 single-cell data (Stuart et al., 2019). A Smart-seq2 count matrix was used to create the Seurat object. Only genes expressed in more than 3 cells were retained. Regarding the cells that were sequenced, only cells with a percentage of mitochondrial genes less than 15% and cells expressing more than 2,000 genes were retained. For trophoblast cells differentiated from human embryos (Zhou et al., 2019), we filtered out cells with the percentage of mitochondrial genes greater than 4% and cells that expressed less than 8,000 genes, and 3,859 trophoblast cells were retained for the subsequent analysis. After the normalization step, we computed highly variable genes with the “mean.var.plot” method. Following scaling all genes in the data, we performed linear dimensional reduction with highly variable genes by default. The “ElbowPlot” function was chosen to determine the dimensionality to perform nonlinear dimensional reduction (UMAP). The graph-based clustering approach was used to cluster the cells by the “FindNeighbors” and “FindClusters” functions. Single-cell data were visualized by the “Dimplot” function. “FeaturePlot,” “VlnPlot,” and “DoHeatmap” functions were used to display the gene expression levels.

### Integrated Analysis of scRNA-seq Data

We used Seurat3 R package with dataset integration function (Stuart et al., 2019) to analyze the trophoblast cell data from hTSCs and trophoblast cells differentiated human embryos (Zhou et al., 2019). After renormalizing the Seurat object, we selected highly variably expressed genes by mean.var.plot method during the FindVariableFeatures step. Then, we performed FindItegarationAnchors and InterateData flows with these highly variable genes and 30 dimensions. After producing the UMAP plot, unsupervised clustering was performed. The DEGs between cell clusters were computed with the Seurat RNA assay.

### DEG Analysis

Highly expressed genes of each cell cluster were analyzed using the Seurat “FindAllMarkers” function on the log-transformed expression matrix. Differentially expressed genes between two cell clusters were found using the Seurat “FindMarkers” function.

### Genotyping PCR and Sequencing

Genomic DNA of wildtype and LMNA^−/−^ hTSCs were extracted using Mouse Direct PCR Kit (B40015, Bimake.com). The targeted region of LMNA sgRNA was amplified using primers LMNA GT-F, GCA​CAG​TAC​CTA​CCA​AGA​GTG​A and LMNA GT-R, AAC​CAA​TCG​AGA​GCA​AGC​AC. The PCR was performed with KOD One PCR Master Mix (TOYOBO) following the instruction. And the targeted mutation was confirmed using Sanger sequencing.

### Lentivirus Production

The packaging of lentivirus Lenti-SpCas9_Puro or Lenti-LMNA sg_Blast was by co-transfecting HEK293T cells with packaging plasmids psPAX2 and pMD2.G. The transfection in HEK293T cells was performed using a polyethyenimine (PEI) transfection protocol (PR40001, Proteintech). The viral supernatants were harvested at 48- and 72 h post transfection, and then filtered through a 0.45 μm PES filter.

### Generation of Monoclonal LMNA^−/−^ hTSCs

2 × 10^4^ hTSCs per well were pre-seeded into a 24-well-plate 24 h before infection. The hTSCs were infected with lentiviruses Lenti-SpCas9_Puro and Lenti-LMNA sg_Blast simultaneously. At 48 h post infection, the infected hTSCs were treated with 2 μg/ml puromycin and 5 μg/ml blasticidin for 48 h. Monoclonal hTSCs were picked for genotyping.

### Quantitative Real-Time PCR

Total RNA was isolated using a RNeasy Mini Kit (Qiagen). cDNA was synthesized using iScript Reverse Transcription Supermix kit (Bio-Rad) and amplified with SYBR Green PCR Master Mix (YEASEN) on a Touch Thermal Cycler Real-Time PCR system (Roche, LightCycler480). GAPDH expression level was used as the internal normalization control. The primers for RNA quantification used in this study as follows:

RT-GAPDH-F, GGA​GCG​AGA​TCC​CTC​CAA​AAT,
RT-GAPDH-R, GGC​TGT​TGT​CAT​ACT​TCT​CAT​GG,
RT-LMNA-F, AAT​GAT​CGC​TTG​GCG​GTC​TAC,
RT-LMNA-R, CAC​CTC​TTC​AGA​CTC​GGT​GAT.

### Statistical Analysis

Images were acquired with 0.65 μm *Z* separation. Three-dimensional visualizations were performed using Imaris. All analyses were carried out using open-source image analysis software, including Zeiss LSM Image Browser Software, Imaris Software, and Fiji Image J (NIH). Imaging data were plotted by GraphPad.

## Results

### Derive Human Trophoblast Stem Cells From Human Blastocysts

**FIGURE 1:**
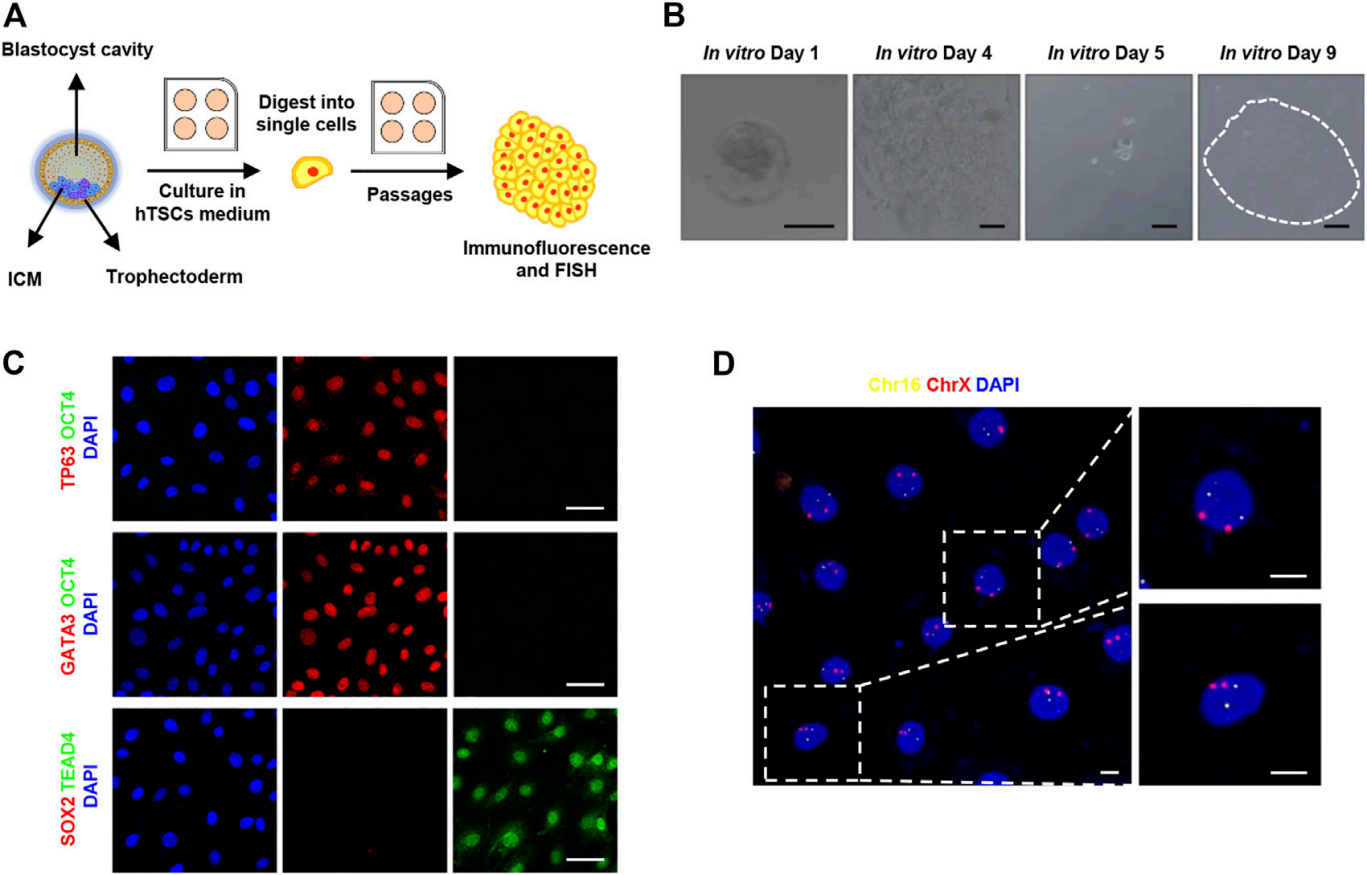
Derivation of human trophoblast stem cells. **(A)** Cartoon depicting the strategy of derivation of human trophoblast stem cells from blastocysts. ICM, inner cell mass; hTSCs, human trophoblast stem cells; FISH, fluorescence *in situ* hybridization. **(B)** Bright-field images showing a human blastocyst and proliferative trophoblast cells. Images from left to right are: Human blastocysts (day 6) were thawed (designated as *in vitro* cultured day 1) and cultured in hTSCs culture medium; human embryos attached to the plates; the attached embryos were digested into single cells; the single cells continued to grow and formed a cell clone. Dotted line shows a cell colony. Scale bars, 100 μm. **(C)** Immunostaining of hTSCs. Markers of trophoblast cells: TP63, TEAD4, and GATA3; markers of embryonic stem cells: OCT4 and SOX2. DAPI, blue, DNA. Scale bars, 50 μm. **(D)** Fluorescence *in situ* hybridization of chromosomes 16 and X in hTSCs. Chr16, chromosome 16, ChrX, chromosome X. DAPI, blue, DNA. Scale bars, 10 μm.

To remodel trophoblast differentiation during implantation, we turned to derive hTSCs as described (Okae et al., 2018) from human blastocysts, as shown in Figure 1A, given the ethical restrictions and the limited number of human embryos available for functional studies. Human blastocysts (day 6) were thawed and placed in 4-well collagen I-coated plates (designated as *in vitro* cultured day 1, IVC day 1) and cultured in hTSCs culture medium. After 2 days of culture, *in vitro* cultured human embryos hatched from zona pellucida and attached to the plates on IVC day 4. Then, the whole attached embryos were digested into single cells and cultured in 4-well collagen I-coated plates for at least 4–5 days. The single cells continued to grow and formed several clones (Figure 1B). The cells in the clone were digested and replated in hTSCs culture medium and cultured until the formation of new clones. After cultured for 10 passages, the proliferative cells were harvested and cultured on Ibidi dishes for immunofluorescent study. To confirm the origination of these cells, we fixed the cells and immunostained them using the lineage markers, as indicated in Figure 1C. These cells highly expressed trophoblast markers, such as TP63, TEAD4, and GATA3, but did not express embryonic stem cell markers, such as OCT4 or SOX2 (Figure 1C). Based on the above immunostaining results, we confirmed these proliferative cells are derived from trophoblast lineage.

In order to exclude cancerous characteristics and confirm the cellular gender, we checked the copy number of chromosomes in these cells, immunofluorescence *in situ* hybridization on chromosomes 16 and X was performed. As shown in Figure 1D, the copy number of chromosomes 16 and X in these cells was 2N, which is consistent with normal somatic cells. It indicates that the established hTSCs carry a diploid set of chromosomes, revealing the hTSCs were karyotypically normal and female.

### The Differentiation Potential of hTSCs

**FIGURE 2:**
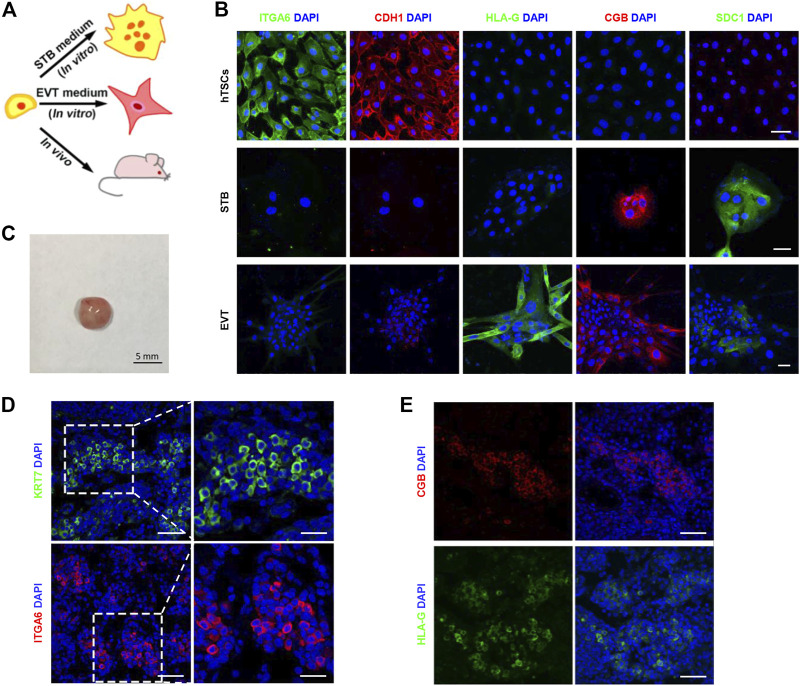
The *in vitro* and *in vivo* differentiation potential of hTSCs. **(A)** Cartoon depicting the differentiation strategy of hTSCs *in vitro* and *in vivo*. STB, syncytiotrophoblast; EVT, extravillous trophoblast cells. **(B)** Differentiation of hTSCs into STB and EVT. Immunostaining of ITGA6 and CDH1 (markers of cytotrophoblast cells); SDC1 and CGB (markers of STB); HLA-G (a marker of EVT). DAPI, blue, DNA. Scale bars, 50 μm. **(C)** Injection of hTSCs (1 × 10^7^) into non-obese diabetic (NOD) severe combined immunodeficiency (SCID) mice. A teratoma from the NOD-SCID mice by day 7. Scale bar, 5 mm. **(D)** Immunostaining of KRT7 (a marker of trophoblast cells) and ITGA6 in a hTSCs-derived lesion. DAPI, blue, DNA. Scale bars, 50 and 20 μm (magnified areas). **(E)** Immunostaining of CGB and HLA-G in a hTSCs-derived lesion. DAPI, blue, DNA. Scale bars, 20 μm.

To test the stemness of these trophoblast cells *in vitro*, we first cultured the human trophoblast stem cells (hTSCs) in the hTSCs medium for 6 days (Figure 2A). Then, we collected the cells and immunostained them for the lineage marker genes. As shown in Figure 2B, the cells were epithelial-like single cells in the presence of hTSCs medium. These cells highly expressed ITGA6 and CDH1 (cytotrophoblast markers) but did not express CGB, SDC1 (STB markers), or HLA-G (an EVT marker). It illustrated the cells in hTSCs medium were maintained in the undifferentiated state. To assess STB-formation capacity of hTSCs, we cultured hTSCs in STB medium for 6 days. In the presence of the STB medium, the cells started to aggregate together and gradually fused to form large syncytia. We fixed and immunostained the cells for linage markers. The STB markers, CGB and SDC1 were highly expressed in these syncytia, whereas ITGA6, CDH1, and HLA-G were poorly expressed (Figure 2B). To evaluate EVT-formation capacity of hTSCs, we cultured hTSCs in EVT medium for 6 days. In the presence of EVT medium, these hTSCs gave rise to mesenchymal-like cells. We fixed and immunostained the cells for linage markers. Cells cultured in EVT medium strongly expressed the EVT marker, HLA-G, on day 6 (Figure 2B). We also detected *CGB*, *SDC1* (markers for STB) and *HLA-G* (a marker for EVT) to identify hTSCs have already differentiated into STB and EVT. It was shown that the expression of *CGB* and *SDC1* significantly increased in hTSCs cultured in STB medium. The expression of *HLA-G* also significantly increased in hTSCs cultured in EVT medium (Supplementary Figure S1A). Overall, these results indicate that hTSCs could differentiate into STB and EVT cells *in vitro*.

Next, we investigated the differentiation potential of hTSCs *in vivo*. We subcutaneously injected the hTSCs (1 ╳ 10^7^) into the back neck of non-obese diabetic (NOD)-severe combined immunodeficiency (SCID) mice (Figure 2A). The injected cells formed 5 mm lesions by day 7 (Figure 2C). Immunostaining of the lesion sections for KRT7 and ITGA6 revealed that the injected cells were CTB-like cells (Figure 2D). To test whether these cells could differentiate into EVT and STB. We immunostained the sections for EVT markers and STB markers. It was identified that the single cells that highly expressed HLA-G as EVT-like cells (Figure 2E; Supplementary Figures S1B,C). CGB-positive STB-like cells were also observed in the lesions (Figure 2E; Supplementary Figures S1B,C). Taken together, expressions of lineage markers demonstrated the pluripotency of hTSCs both *in vitro* and *in vivo*.

**FIGURE 3:**
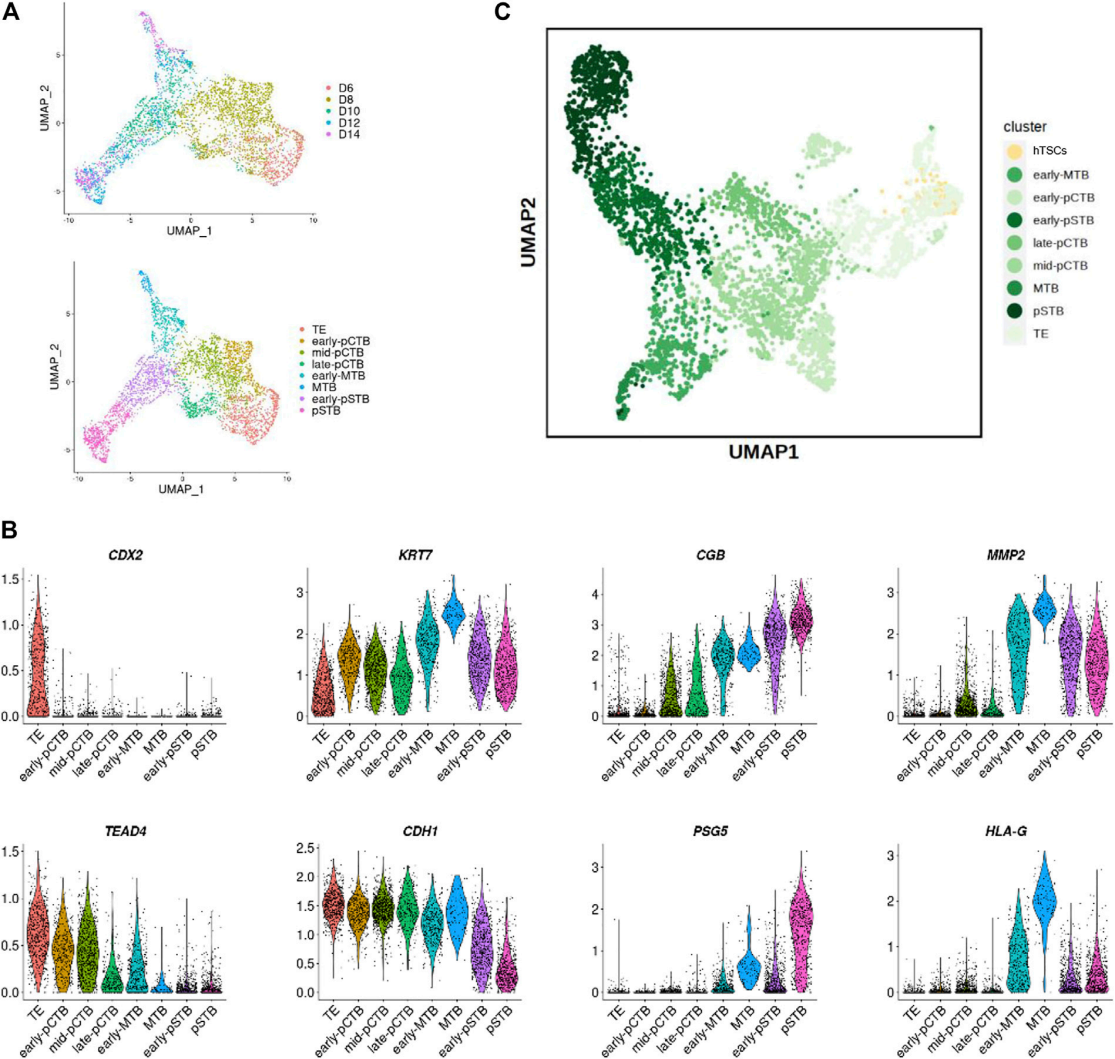
Developmental matching of hTSCs with peri-implantation trophoblast cells of human embryos. **(A)** UMAP plots showing the expression patterns of trophoblast cells in human embryos. Upper, different time points; Lower, different clusters. **(B)** Violin plots showing the expression patterns of marker genes. **(C)** Joint visualization of hTSCs together with trophoblast cells from published datasets of human embryos cultured *in vitro*.

We then sought to characterize the molecular signature of hTSCs. To this end, we performed scRNA-seq for selected hTSCs. We then compared hTSCs with published datasets from cultured human embryos (Zhou et al., 2019). Trophoblast cells from human embryos were clearly ordered according to the embryonic day (Supplementary Figure S2A). To explore the cellular composition of these trophoblast cells, we performed differential expression gene (DEG) analysis to define each cell cluster. The cells were divided into 8 clusters, including TE, 3 clusters of primitive cytotrophoblast cells (pCTB), 2 clusters of migrative trophoblast cells (MTB), 2 clusters of primitive STB (pSTB) according to lineage marker genes, such as *CDX2*, *TEAD4*, *KRT7*, *CDH1*, *CGB*, *PSG5*, *MMP2*, and *HLA-G*, and developmental time (Figures 3A,B; Supplementary Figure S2B). Comparative transcriptome analysis showed our blastocyst-derived hTSCs were close in the gene signature with the TE cells of the human blastocyst (Figure 3C).

### Nuclei Enlargement in Syncytialization of hTSCs

The main feature of pSTB after implantation is nuclear enlargement as described by Carnegie Collection. We next investigated the molecular pathways that could be responsible for this critical morphology. Given the ethical restrictions and the limited number of human embryos available for loss/gain-of-function studies, we utilized the established hTSCs as described above to remodel trophoblast morphogenesis during implantation. First, to determine whether the STB differentiated from hTSCs mimic nuclei enlargement, we cultured hTSCs in hTSCs medium and STB medium for 5 days, respectively, and performed immunofluorescence (IF) analysis with Lamin A (a nuclear envelope protein), CGB (a marker for STB) and F-actin antibodies. We found that CGB^+^ STB exhibited obvious nuclear enlargement when compared with hTSCs (Supplementary Figure S3A). To quantify the nuclear volume in hTSCs and STB, we 3D reconstructed the nuclei based on the staining results of hTSCs and STB. We found the nuclei in STB were considerably enlarged when compared with those in mononucleated hTSCs (Supplementary Figure S3B). Taken together, these results demonstrated that syncytialization of hTSCs recapitulated nuclear enlargement of trophoblast development in human embryos.

### CRISPR-Cas9 Genome Editing of the *LMNA* Gene in the hTSCs

**FIGURE 4:**
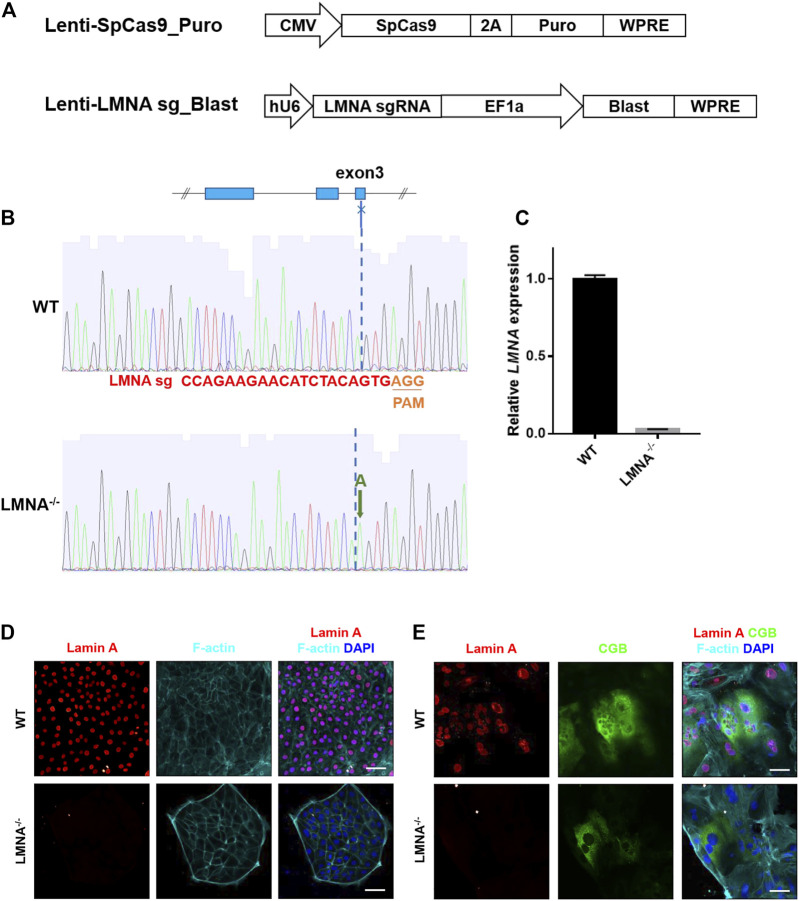
Construction of LMNA^−/−^ hTSCs. **(A)** The lentiviral vectors used for SpCas9 (the top penal) and LMNA sgRNA (the bottom panel) expression. CMV, human cytomegalovirus immediate early promoter; EF1a, elongation factor-1α core promotor; hU6, RNA polymerase III promotor for human U6 SnRNA; 2A, 2A self-cleaving peptide; Puro, puromycin selection marker; Blast, blasticidin selection marker; WPRE, posttranscriptional regulatory element. **(B)** Sanger sequencing results of the LMNA sgRNA target site in wildtype (WT) and LMNA^−/−^ hTSCs. The LMNA sgRNA used in this study targets to the third exon of *LMNA* gene. **(C)** qRT-PCR analysis of LMNA mRNA expression in WT and LMNA^−/−^ hTSCs. Graph showing the expression level relative to the geometric mean of the housekeeping gene GAPDH. Data are shown as mean ± s.e.m. **(D)** Immunostaining of WT and LMNA^−/−^ hTSCs for Lamin A and F-actin. DAPI, blue, DNA. Scale bars, 50 μm. **(E)** Immunostaining of WT and LMNA^−/−^ STB for Lamin A, CGB, and F-actin. DAPI, blue, DNA. Scale bars, 50 μm.

Lamin A has been strongly implicated in anchoring heterochromatin to the nuclear periphery in multiple cell types and functions as a regulator of nuclear shape (Solovei et al., 2013). To investigate the role of lamin A in the regulation of nuclear enlargement during syncytialization, we performed a targeted disruption of *LMNA* in hTSCs using the clustered regularly interspaced short palindromic repeats (CRISPR)-Cas9 system. *LMNA* gene has several splicing products, including Lamin A, Lamin C, Lamin A delta 10, and Lamin C2 (Solovei et al., 2013). We designed a single-guide-RNA (sgRNA) targeting the third exon of *LMNA* gene coding sequence (*LMNA* sgRNA), which was involved in the common region of the main transcripts. SpCas9 and LMNA sgRNA were respectively packaged into two lentiviruses, and introduced into hTSCs (Figure 4A). The hTSCs carrying both SpCas9 and targeting sgRNA were obtained by simultaneously enrichment of their antibiotic selection markers, and monoclonal hTSC was picked for genotyping (Figure 4B). Sanger sequencing of the target region from the resulting hTSC revealed a successful insertion of adenine (A) into both alleles (Figure 4B). Edited hTSC remained normal cell morphology (Supplementary Figure S4A). Quantitative real time PCR (qRT-PCR) detection of the *LMNA* mRNA in the edited hTSC showed that the mutation caused a non-sense-mediated RNA decay (Figure 4C). Immunostaining of *LMNA* in the edited hTSCs confirmed the inhibition of LMNA protein expressions (Figure 4D). These studies confirmed the loss of *LMNA* in hTSC, and the edited hTSC was designated LMNA^−/−^ hTSC. To further investigate the role of *LMNA* in STB, we differentiated LMNA^−/−^ hTSC to STB. Immunostaining of LMNA protein in the STB revealed that the STB lost the expression of *LMNA* protein (Figure 4E).

### Loss of *LMNA* Inhibited the Fusion Ability of hTSCs

**FIGURE 5:**
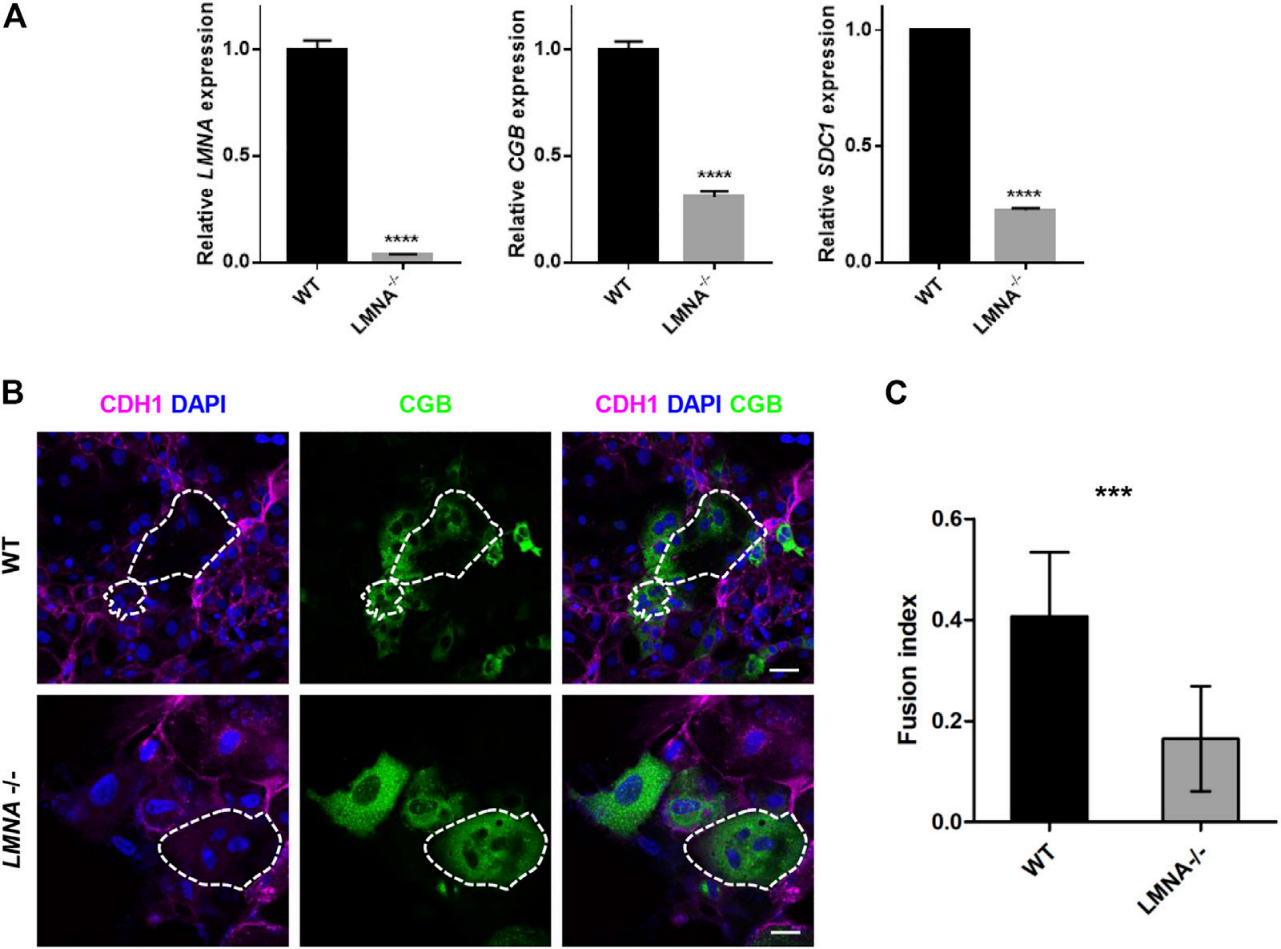
Loss of LMNA inhibited the fusion ability of hTSCs. **(A)** qRT-PCR analysis of LMNA, CGB, SDC1 mRNA expression in WT and LMNA^−/−^ hTSCs. Graph showing the expression level relative to the geometric mean of the housekeeping gene GAPDH. Data are shown as mean ± s.e.m. **(B)** Immunostaining of WT and LMNA^−/−^ STB for CDH1and CGB. Dotted area represented multinucleated STB. DAPI, blue, DNA. Scale bars, 50 μm. **(C)** Quantification of fusion index in WT and LMNA^−/−^ STB. *n* = 10 fields of view, three experiments. Data are shown as mean ± s.e.m. Unpaired two-tailed Student’s t-test, ***p < 0.001. The fusion index was determined by N-S/T, where N was the number of nuclei in STB, S was the number of STB and T was the number of the total number of nuclei.

To examine the function of LMNA during syncytialization, we firstly culture the WT and LMNA^−/−^ hTSCs in the STB medium for 6 days with the same initial density. Then, we detected the RNA level of *LMNA*, *CGB*, and *SDC1* in WT and LMNA^−/−^ hTSCs. We found that loss of *LMNA* inhibited the expression of *CGB* and *SDC1* in hTSCs cultured in STB medium on day 6 (Figure 5A). To further confirm the function of LMNA in inhibiting syncytialization, we culture the WT and LMNA^−/−^ hTSCs in the STB medium for 6 days with the same initial density. By day 6, we performed immunofluorescence analyses with CDH1 (a marker for mononucleated trophoblast cells) and CGB (a marker for syncytiotrophoblast) antibodies. STB was formed in wild-type (WT) hTSCs as evidenced by the appearance of multiple nuclei within the cytoplasm. However, LMNA^−/−^ hTSCs were rarely composed of multinucleated syncytia (Figure 5B). We then detected the fusion index based on the immunofluorescence results. The percentages of multinucleated syncytia were 40.7% and 16.5% in WT and LMNA^−/−^ hTSCs, respectively (Figure 5C), indicating in the loss of LMNA, the number of multinucleated syncytia decreased. We also used GFP-hTSCs as a control group to exclude the influence of genetic manipulation. The fusion index did not show significant difference in GFP-hTSCs and WT hTSCs (Supplementary Figures S5A–C). Taken together, these results demonstrated that loss of LMNA gene inhibited the fusion ability of hTSCs.

### Loss of *LMNA* Promoted Nuclear Enlargement During Syncytialization

**FIGURE 6:**
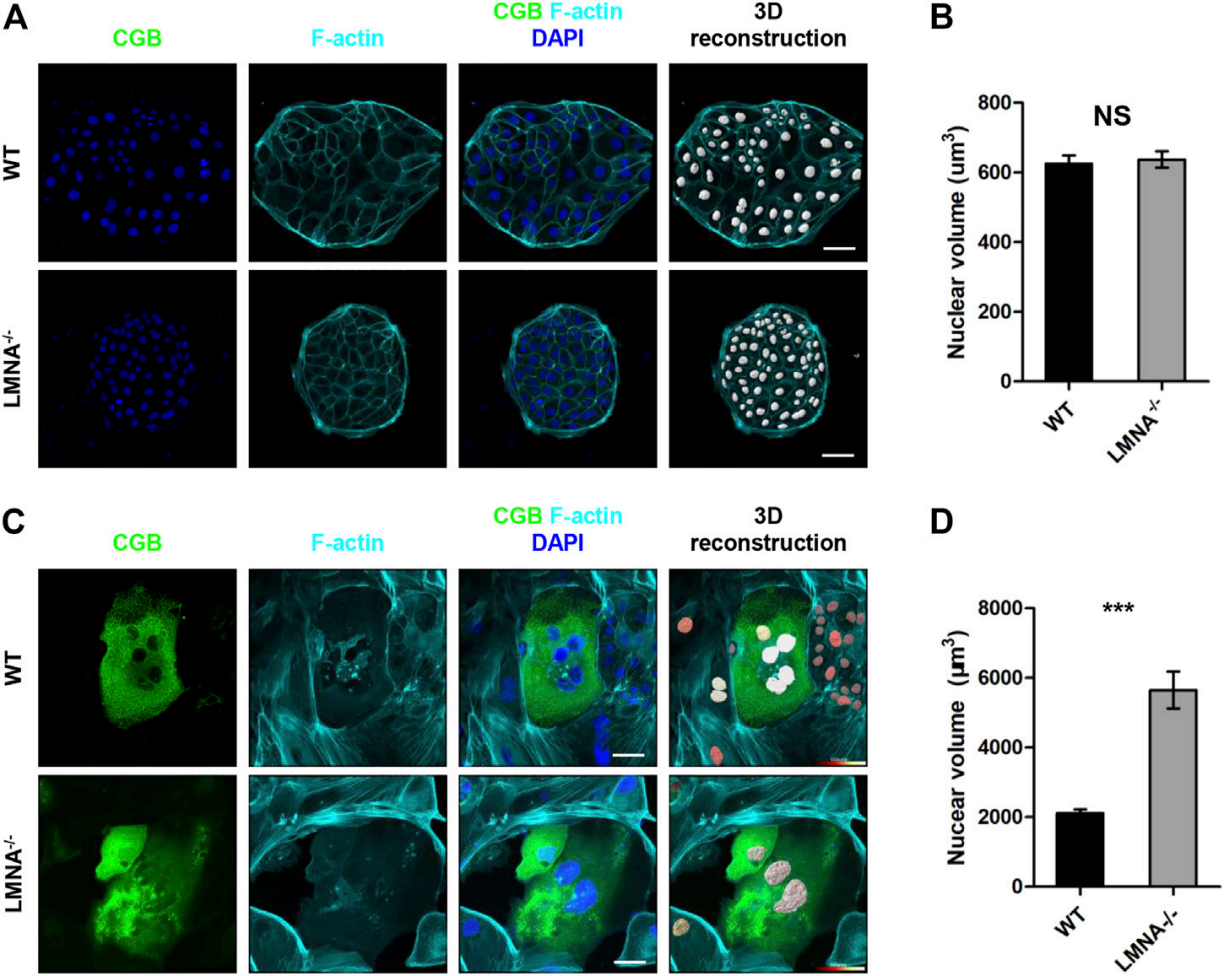
Loss of LMNA promotes nuclear enlargement during syncytialization. **(A)** Immunostaining of WT and LMNA^−/−^ hTSCs for F-actin and CGB. DAPI, blue, DNA. Scale bars, 50 μm. The nuclei were 3D reconstructed. **(B)** Quantification of nuclear volume in WT and LMNA^−/−^ hTSCs. *n* = 10 fields of view, three experiments. Data are shown as mean ± s.e.m. Unpaired two-tailed Student’s t-test, NS, not significant. **(C)** Immunostaining of WT and LMNA^−/−^ STB for CGB and F-actin. DAPI, blue, DNA. Scale bars, 50 μm. The nuclei were 3D reconstructed. Color key from red to white indicated nuclear volume levels from low to high. **(D)** Quantification of nuclear volume in WT and LMNA^−/−^ STB. *n* = 10 fields of view, three experiments. Data are shown as mean ± s.e.m. Unpaired two-tailed Student’s t-test, ***p < 0.001.

Mesh-structured nuclear lamin A is well known to modulate nuclear shape via remodeling of actin cytoskeleton in a two-dimensional cell culture (Dahl et al., 2008; Hale et al., 2008). We hypothesized that lamin A might also be a factor contributing to the dynamic changes of nuclear morphology during syncytialization. Firstly, we cultured the WT and LMNA^−/−^ hTSCs in the hTSCs medium. The nuclei were reconstructed basing on the IF of DAPI (Figure 6A). The nuclear volume of WT and LMNA^−/−^ hTSCs had no significant changes (Figure 6B). We then cultured the WT and LMNA^−/−^ hTSCs in the STB medium for 6 days in the same initial density. By day 6, we performed immunofluorescence analyses with and CGB and F-actin antibodies. To quantitatively measure the 3D morphology of the nucleus, we analyzed 3D-reconstructed confocal images of the nucleus (Figure 6C). Depth-dependent color-coded 3D-rendering of the nuclear surface distinguished the nuclear morphology in WT STB from LMNA^−/−^ STB (Figure 6C). We then calculated the nuclear volume basing on 3D-nuclear morphology of wild-type (WT) hTSCs and LMNA^−/−^ hTSCs cultured in STB medium. In LMNA^−/−^ STB, the average nuclear volume was larger than that of WT STB (Figure 6D). Overall, these net results indicated that *LMNA* potentially has an intrinsic role in the regulation of hTSCs syncytialization, including contributing to the construction of 3D-nuclear morphology during syncytialization.

## Discussion

Implantation of the blastocyst is a milestone event in human embryonic development (Larsen, 1974; Aplin, 2000). Primary syncytialization is vitally important towards implantation. Optimal primary syncytialization involves morphologically identifiable features such as nuclear enlargement. However, due to limited numbers of human embryos and ethical issues for genetic manipulation of human embryos, the underlying mechanism of trophoblast differentiation, especially nuclear enlargement, during implantation is yet to be determined. Here, we have established human trophoblast stem cells (hTSCs) from human blastocysts. We identified nuclear enlargement in syncytiotrophoblast differentiated from hTSCs, reminiscent of primary syncytialization that physiologically occurs during human embryo implantation. Moreover, we set up hTSCs and *LMNA* loss of function hTSCs models and revealed critical roles for lamin layer in reducing the fusion ability of hTSCs and enlarging the nuclear volume. In short, syncytialization of hTSCs can morphologically remodel primary syncytialization of human embryos and serve as a platform to investigate the mechanism of primary syncytialization during implantation.

Primary syncytialization is an essential event that leads to the implantation of embryos. Studies on primary syncytialization during implantation have been largely restricted to sections of the Carnegie Collection (Hertig et al., 1956). Prior studies have noted the urgent need for an *in vitro* model to mimic this situation during implantation. Commonly *in vitro* models are choriocarcinoma cell-derived spheroids (Rothbauer et al., 2017) and human embryonic stem cells (hESCs) derived trophoblast-like cells (Xu et al., 2002; Yue et al., 2020). However, choriocarcinoma cells possess cancer features and the transcriptome is different from those of primary trophoblast cells. Trophoblast-like cells derived from hESCs resemble human trophectoderm during implantation but not subsequent syncytiotrophoblast. Nowadays, the system of culturing human embryos to post-implantation stage is also established. However, owing to the limited number of human embryos available and ethical issues for genetic manipulation, it is necessary to obtain a model to investigate the trophoblast development during implantation. Recent developments in hTSCs that cultured atop a two-dimensional (2D) surface have fulfilled the need for trophoblast specification (Okae et al., 2018). However, whether hTSCs could be used for investigating trophoblast differentiation during implantation remains an open question. In this study, hTSCs have been derived from human blastocysts. Multiple differentiation potentials of hTSCs have also been proved by *in vivo* and *in vitro* experiments. The derivation of hTSCs could help to investigate trophoblast differentiation during implantation.

The nuclear morphology changes in various severe genetic disorders, collectively termed laminopathies, are attributed to the abnormalities in the nuclear lamina mainly caused by the mutation of *LMNA* (Hutchison, 2002; Schreiber and Kennedy, 2013). On the basis of our findings, it has been suggested that the changes in nuclear shape are intimately linked to syncytialization. The results we present here further indicated that *LMNA* may control the nuclear volume during syncytialization. The cell fusion efficiency was hampered by knocking out *LMNA* and the enlarged nuclei were increased. While our data highlights the possible function of *LMNA* in controlling nuclear morphology, cell nucleus also serves as a mechanotransducer (Enyedi et al., 2016). Multiple biophysical signals are transmitted from the cytoskeleton to intranuclear chromosomal realignment of the cell body. Leveraging the hTSCs, it would be important in future studies to determine the relationship between extracellular mechanical stimuli and intracellular responses.

In conclusion, our findings support that STB differentiated from hTSCs can morphologically simulate nuclear enlargement in primary syncytialization of human embryos. Remarkably, our study provides a valuable platform for indicating physiologic significance of *LMNA* in nuclear enlargement. Finally, we demonstrated that *in vitro* cultured human embryos and hTSCs are of great interest as a model system for studying cellular and molecular mechanisms of the mysterious and vital stage of trophoblast development as well as illustrating the related pathologies in early pregnancy.

## Data Availability

The datasets presented in this study can be found in online repositories. The names of the repository/repositories and accession number(s) can be found below: https://www.ncbi.nlm.nih.gov/geo/query/acc.cgi?acc=GSE165131. Gene expression omnibus GSE165131.
