# Supplementary material for: LMNA Determines Nuclear Morphology During Syncytialization of Human Trophoblast Stem Cells
Source: Front Cell Dev Biol. 2022 Apr 11;10:836390. doi: 10.3389/fcell.2022.836390 (PMC9035786; doi:10.3389/fcell.2022.836390)
Supplement: Supplementary file 1 [file DataSheet1.pdf]

Supplementary Materials for

LMNA determines nuclear morphology during syncytialization of human trophoblast stem cells

This PDF file includes:

Supplementary figure 1 to 5
Supplementary Text
Supplemental Code

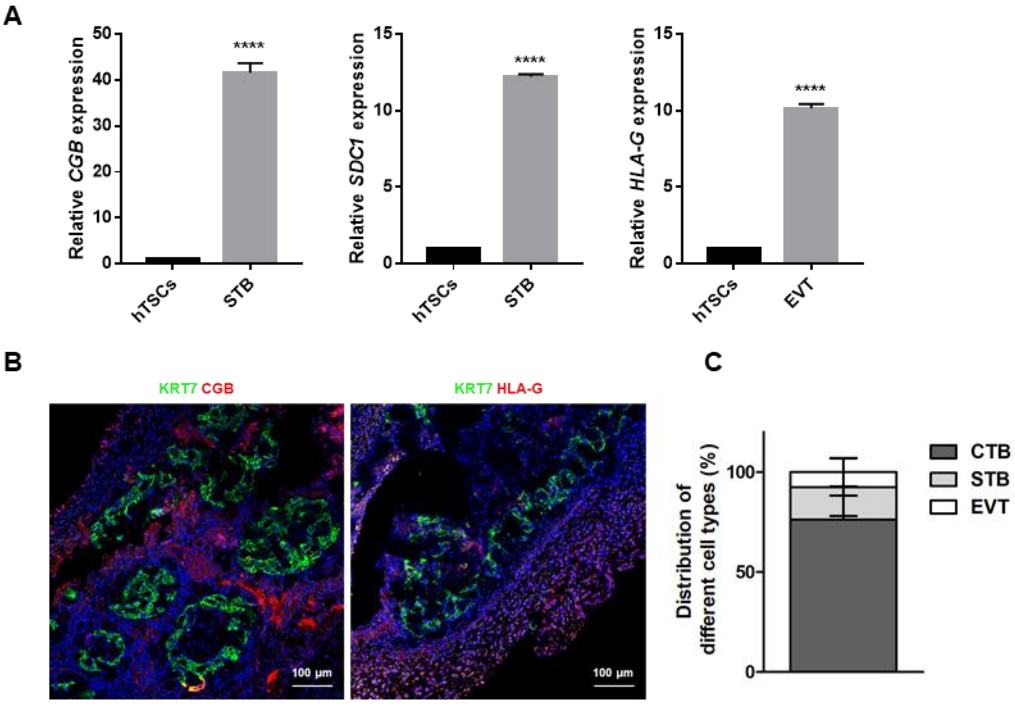

Supplementary figure 1. The differentiation potential of hTSCs *in vitro* and *in vivo*.

(A) qRT-PCR analysis of CGB, SDC1, HLA-G mRNA expression in hTSCs, STB, and EVT. Graph showing the expression level relative to the geometric mean of the housekeeping gene GAPDH. Data are shown as mean ± s.e.m..

(B) Immunostaining of KRT7, CGB, and HLA-G in a hTSCs-derived lesion. DAPI, blue, DNA. Scale bars, 100 μm.

(C) Bar graph showing the proportion of different cell types in hTSCs-derived lesion. Data are shown as mean ± s.e.m.. CTB, cytotrophoblast; STB, syncytiotrophoblast; EVT, extravillous trophoblast cells.

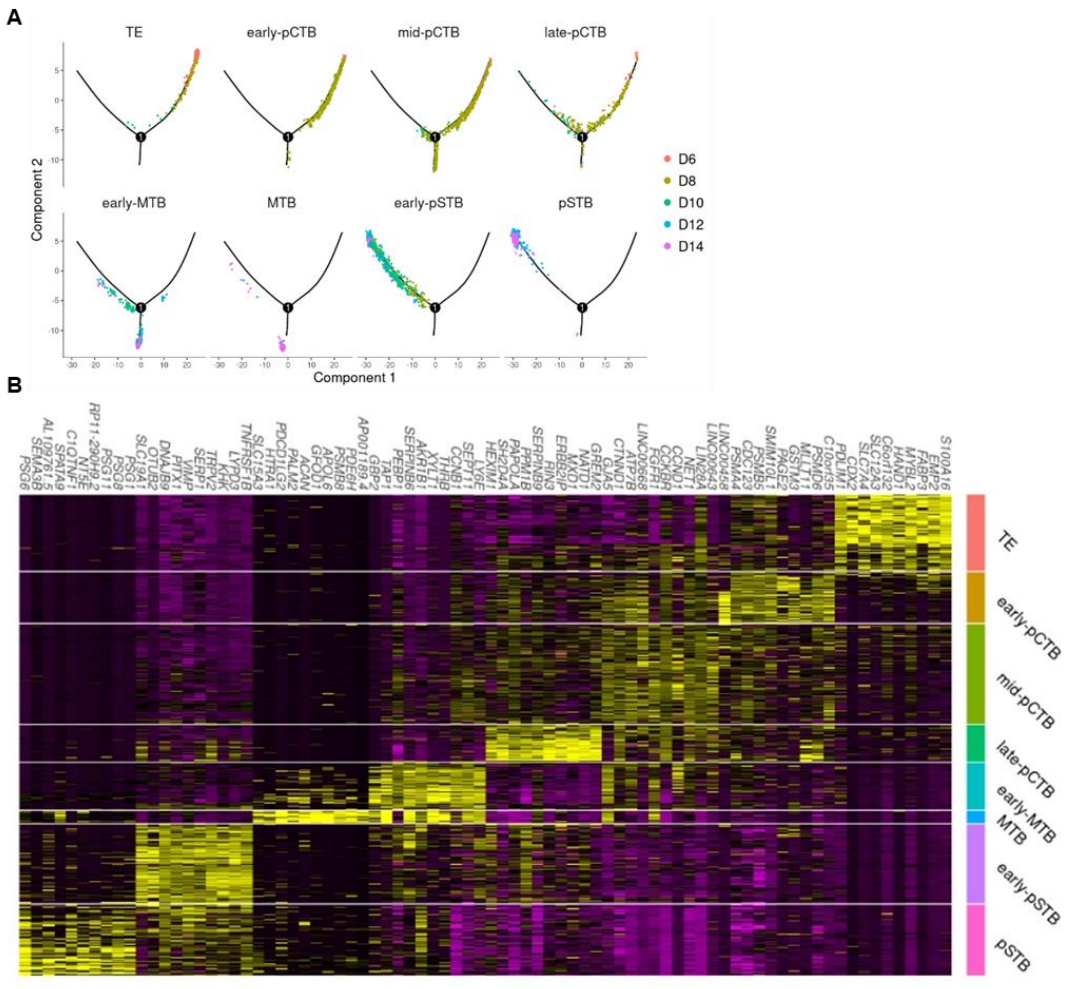

Supplementary figure 2. Developmental matching of hTSCs with peri-implantation trophoblast cells of human embryos.

(A) Pseudotime analysis showing embryonic stage and lineage assignment. Three-dimensional diffusion map representation of all cells at different time points of culture, showing lineage assignment on each embryonic day.

(B) Heatmap showing the representative DEGs in different clusters of human trophoblast cells.

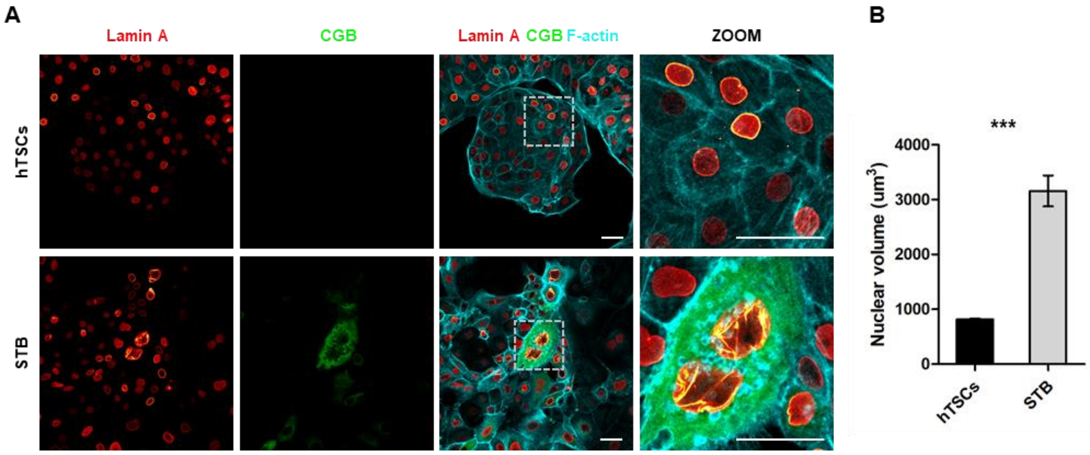

Supplementary figure 3. Nuclear enlargement in syncytiotrophoblast.

(A) Immunostaining of hTSCs and STB for Lamin A, CGB, and F-actin. Dotted rectangle represented regions that are shown with higher magnification. Scale bars, 50 μm.

(B) Quantification of nuclear volume in hTSCs and STB. n = 10 fields of view, three experiments. Data are shown as mean ± s.e.m.. Unpaired two-tailed Student's t-test, ***P<0.001.

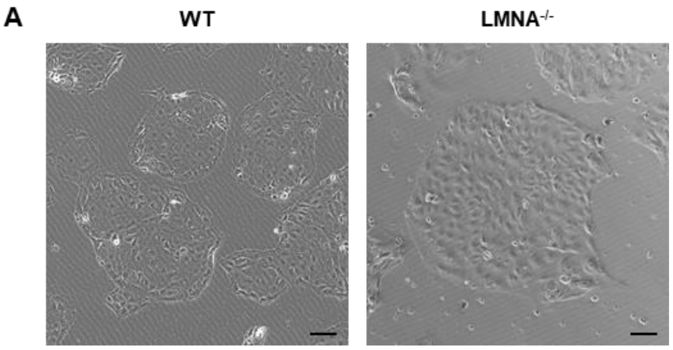

Supplementary figure 4. Morphology of WT and LMNA$^{-/-}$ hTSCs.
(A) Bright-field images of WT and LMNA$^{-/-}$ hTSCs. Scale bars, 100 μm.

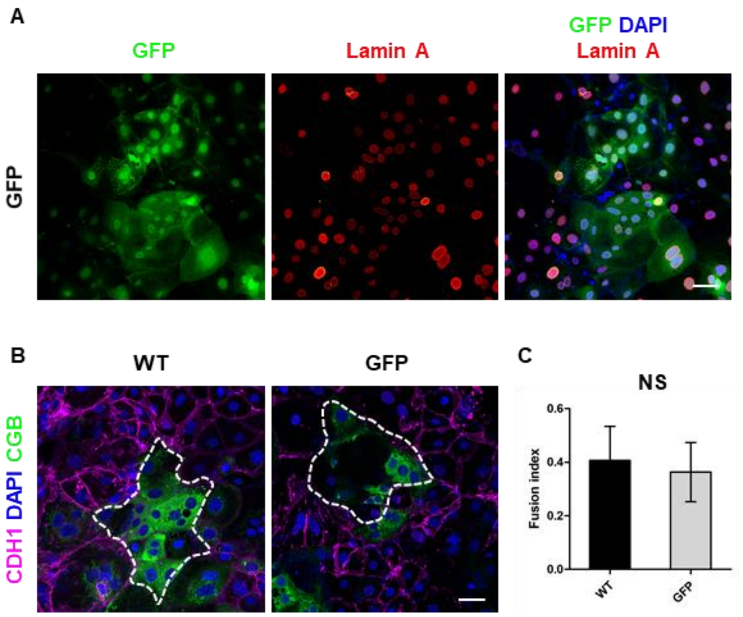

Supplementary figure 5. The syncytialization of GFP-hTSCs.

(A) Immunostaining of GFP-hTSCs for Lamin A. DAPI, blue, DNA. Scale bars, 50 μm.

(B) Immunostaining of WT and GFP-STB for CDH1 and CGB. Dotted area represented multinucleated STB. DAPI, blue, DNA. Scale bars, 50 μm.

(C) Quantification of fusion index in WT and GFP-STB. n = 10 fields of view, three experiments. Data are shown as mean ± s.e.m.. Unpaired two-tailed Student's t-test, NS, not significance.

Supplementary Text

Competing financial interests

The authors declare no competing financial interests.

Materials & Correspondence

Correspondence and material requests should be addressed to Hongmei Wang.

Supplemental Code: cncjgmianlitvkf
